# Supplementary figures and images for: Genome-wide association study between SARS-CoV-2 single nucleotide polymorphisms and virus copies during infections
Source: PLoS Comput Biol. 2024 Sep 17;20(9):e1012469. doi: 10.1371/journal.pcbi.1012469 (PMC11432881; doi:10.1371/journal.pcbi.1012469)

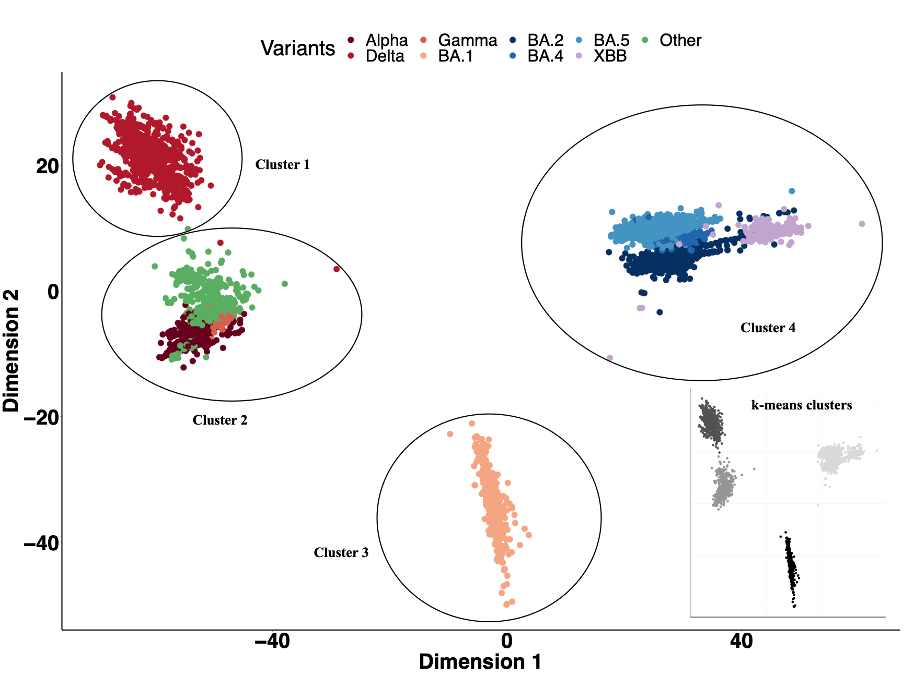

Supplement: S1 Fig — Clusters are defined using a k-means clustering method, as demonstrated on the bottom right corner. (TIF) [file pcbi.1012469.s001.tif]

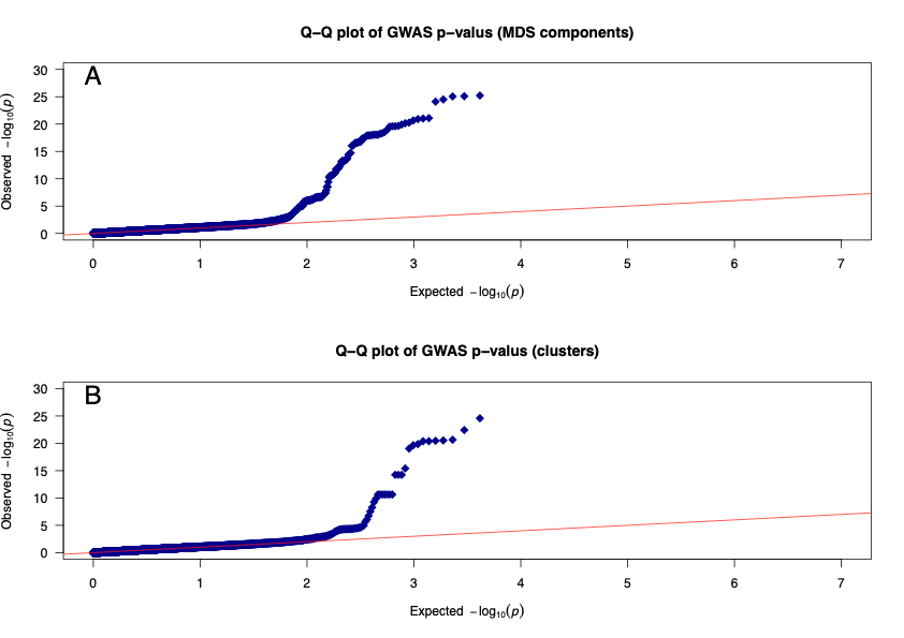

Supplement: S2 Fig — Q-Q plots (quantile-quantile plots) showing the p-values from GWAS analysis using (A) two MDS-computed components, or (B) MDS-inferred four clusters as covariates in the regression model. (TIF) [file pcbi.1012469.s002.tif]

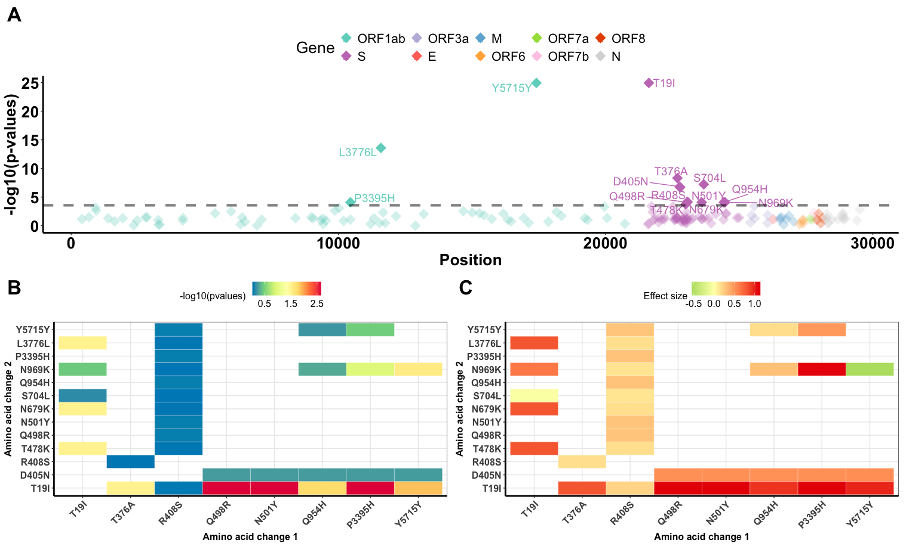

Supplement: S3 Fig — (A) Marginal epistasis test results of the SNPs (annotated as amino acid changes) that have marginal epistatic effects on viral copies. The dashed line indicates the permuted threshold for genome-wide significance p = 0.05/171 = 2.74×10^−4^. Significant mutations are shown with solid colors. (B) The p-values and (C) the effect size of pairwise interaction tests among the significant mutations. (TIF) [file pcbi.1012469.s003.tif]

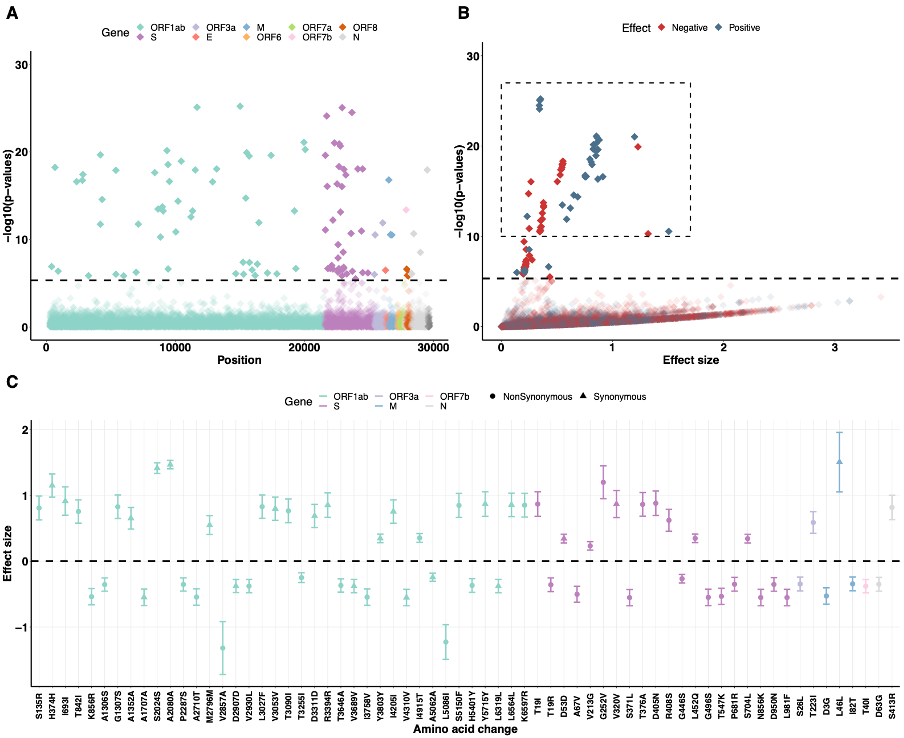

Supplement: S4 Fig — (A) Genome-wide association results of the impact of identified SNPs on viral copies during SARS-CoV-2 infection. The dashed line indicates the permuted threshold for genome-wide significance p = 4.67×10^−6^. Significant SNPs are shown with solid colors. (B) SNPs (with p<1×10^−10^) that have positive (blue) or negative (red) effects on viral copies. (C) The corresponding synonymous (triangles) and non-synonymous (circles) amino acid changes that associate with increased or decreased viral copies. Data is shown as means with 95% confidence intervals. The estimated effective sizes and associated standard deviations are given in S1 Table. A Q-Q plot showing the observed distribution of p-value and the expected distribution is given in S2 Fig. (TIF) [file pcbi.1012469.s004.tif]

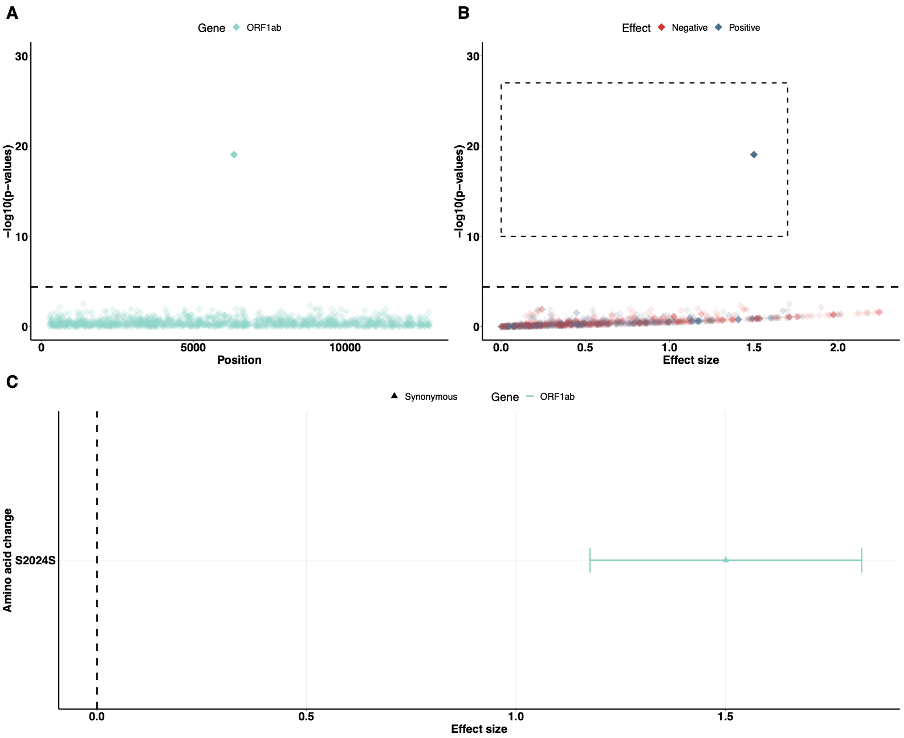

Supplement: S5 Fig — (A) Genome-wide association results of the impact of identified SNPs on viral copies during SARS-CoV-2 infection. The dashed line indicates the permuted threshold for genome-wide significance p = 4.03×10^−5^ (0.05/1242 SNPs). Significant SNPs are shown with solid colors. (B) SNPs (with p<1×10^−10^) that have positive (blue) or negative (red) effects on viral copies. (C) The corresponding synonymous (triangles) amino acid changes that associate with increased or decreased viral copies. Data shown as means with 95% confidence intervals. (TIF) [file pcbi.1012469.s005.tif]

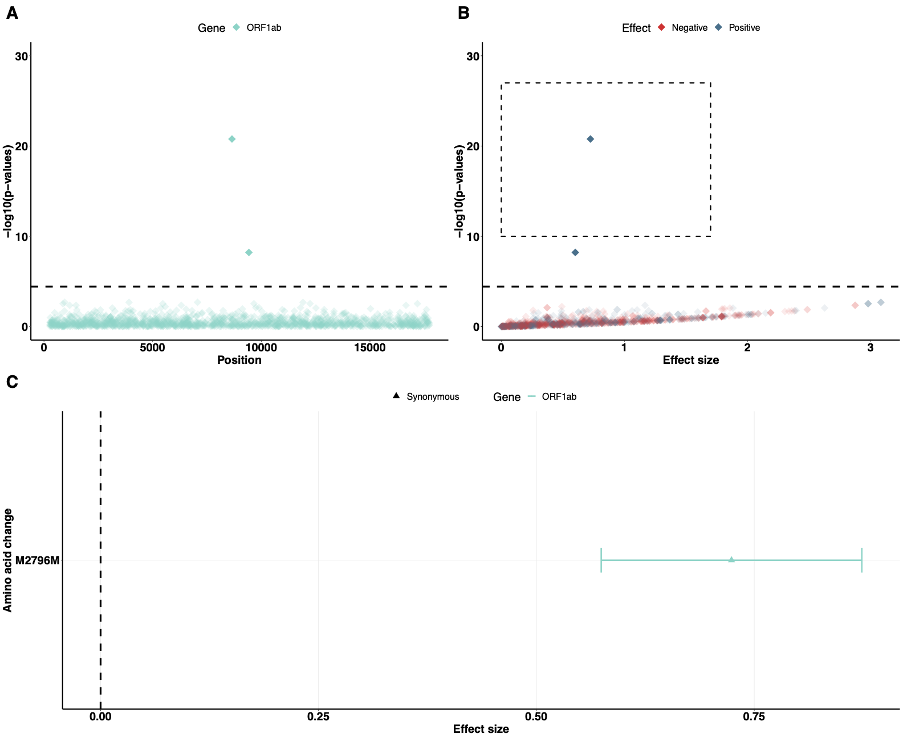

Supplement: S6 Fig — (A) Genome-wide association results of the impact of identified SNPs on viral copies during SARS-CoV-2 infection. The dashed line indicates the permuted threshold for genome-wide significance p = 3.68×10^−5^(0.05/1357 SNPs). Significant SNPs are shown with solid colors. (B) SNPs (with p<1×10^−10^) that have positive (blue) or negative (red) effects on viral copies. (C) The corresponding synonymous (triangles) amino acid changes that associate with increased or decreased viral copies. Data shown as means with 95% confidence intervals. (TIF) [file pcbi.1012469.s006.tif]

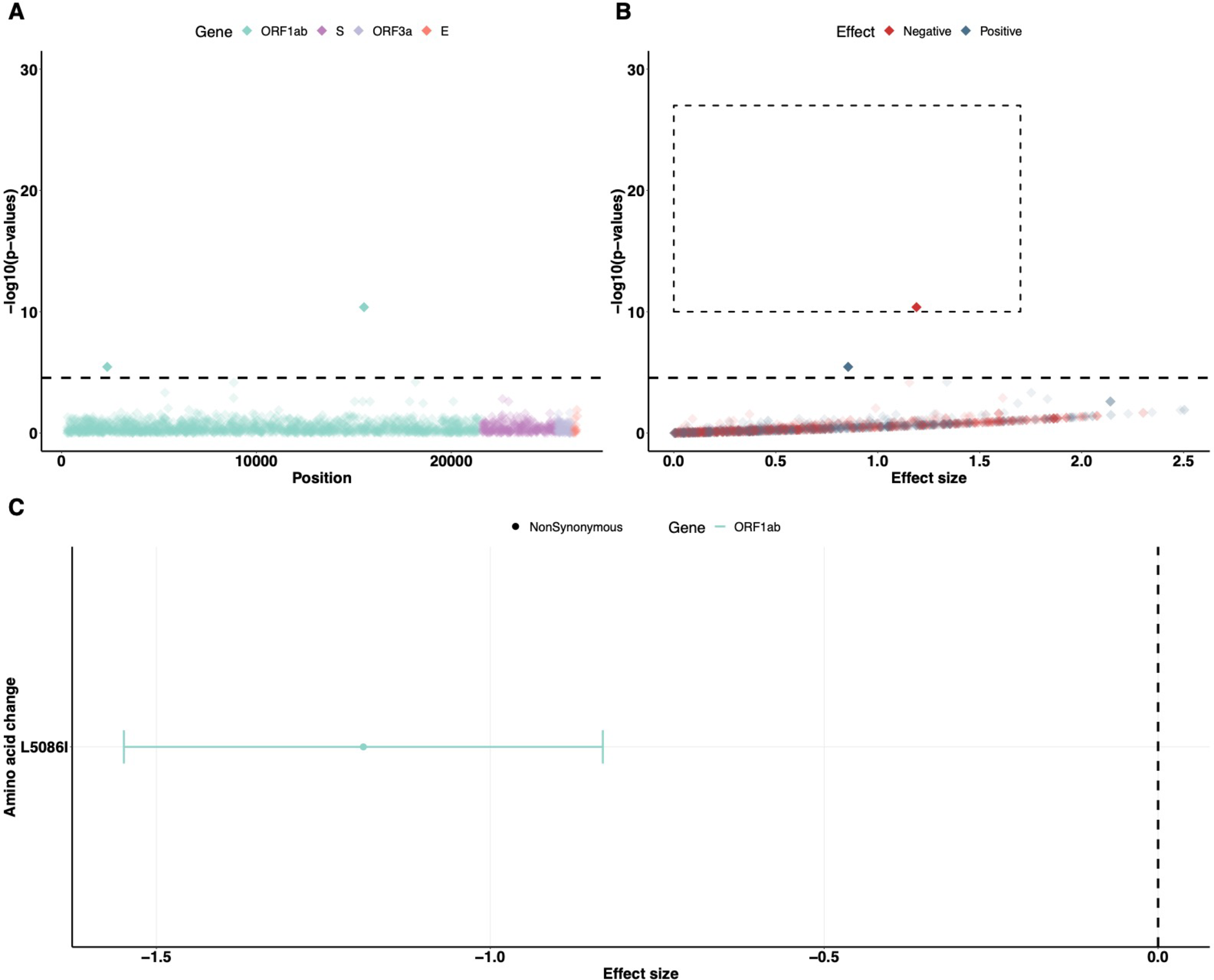

Supplement: S7 Fig — (A) Genome-wide association results of the impact of identified SNPs on viral copies during SARS-CoV-2 infection. The dashed line indicates the permuted threshold for genome-wide significance p = 2.80×10^−5^(0.05/1784 SNPs). Significant SNPs are shown with solid colors. (B) SNPs (with p<1×10^−10^) that have positive (blue) or negative (red) effects on viral copies. (C) The corresponding non-synonymous (circles) amino acid changes that associate with increased or decreased viral copies. Data shown as means with 95% confidence intervals. (TIF) [file pcbi.1012469.s007.tif]

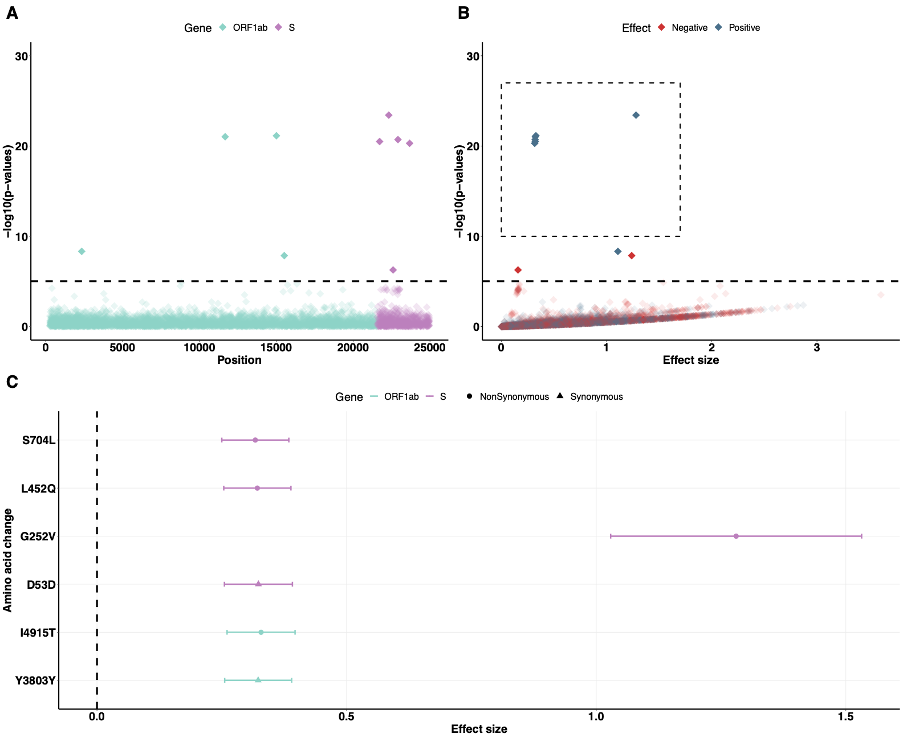

Supplement: S8 Fig — (A) Genome-wide association results of the impact of identified SNPs on viral copies during SARS-CoV-2 infection. The dashed line indicates the permuted threshold for genome-wide significance p = 7.91×10^−6^ (0.05/6314 SNPs). Significant SNPs are shown with solid colors. (B) SNPs (with p<1×10^−10^) that have positive (blue) or negative (red) effects on viral copies. (C) The corresponding synonymous (triangles) and non-synonymous (circles) amino acid changes that associate with increased or decreased viral copies. Data shown as means with 95% confidence intervals. (TIF) [file pcbi.1012469.s008.tif]

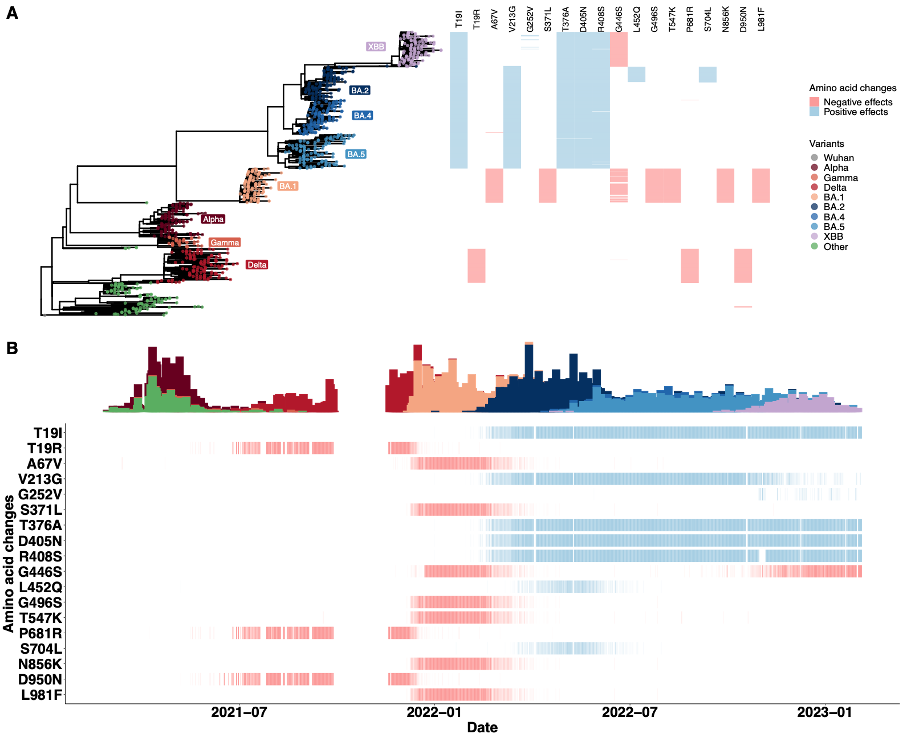

Supplement: S9 Fig — The results are based on the multivariate regression analysis using the two MDS components as covariates. (A) The phylogenetic tree estimated from a representative set of 996 genome sequences showing variant assignments and the locations of amino acid changes that increase (blue) or decrease (red) viral copies. (B) The temporal dynamics of the SNPs from February 2021 to March 2023. The transparency of the color corresponds to the mutation fraction in the daily sequence count: transparent color indicates low fractions, and opaque color indicates high fractions. (TIF) [file pcbi.1012469.s009.tif]

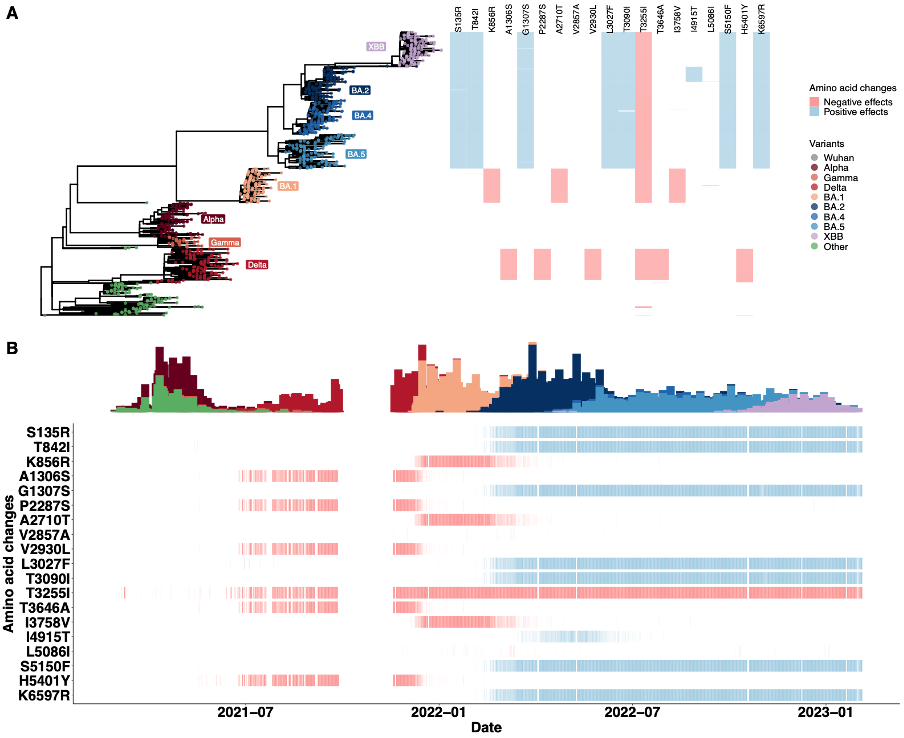

Supplement: S10 Fig — The results are based on the multivariate regression analysis using the two MDS components as covariates. (A) The phylogenetic tree estimated from a representative set of 996 genome sequences showing variant assignments and the locations of amino acid changes that increase (blue) or decrease (red) viral copies. (B) The temporal dynamics of the SNPs from February 2021 to March 2023. The transparency of the color corresponds to the mutation fraction in the daily sequence count: transparent color indicates low fractions, and opaque color indicates high fractions. (TIF) [file pcbi.1012469.s010.tif]

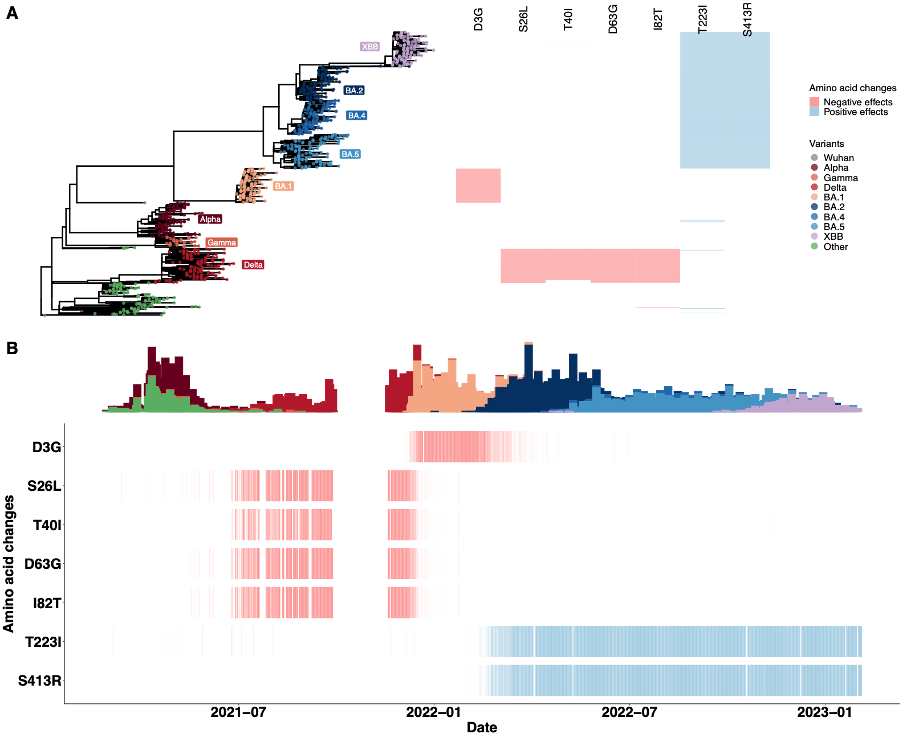

Supplement: S11 Fig — The results are based on the multivariate regression analysis using the two MDS components as covariates. (A) The phylogenetic tree estimated from a representative set of 996 genome sequences showing variant assignments and the locations of amino acid changes that increase (blue) or decrease (red) viral copies. (B) The temporal dynamics of the SNPs from February 2021 to March 2023. The transparency of the color corresponds to the mutation fraction in the daily sequence count: transparent color indicates low fractions, and opaque color indicates high fractions. (TIF) [file pcbi.1012469.s011.tif]

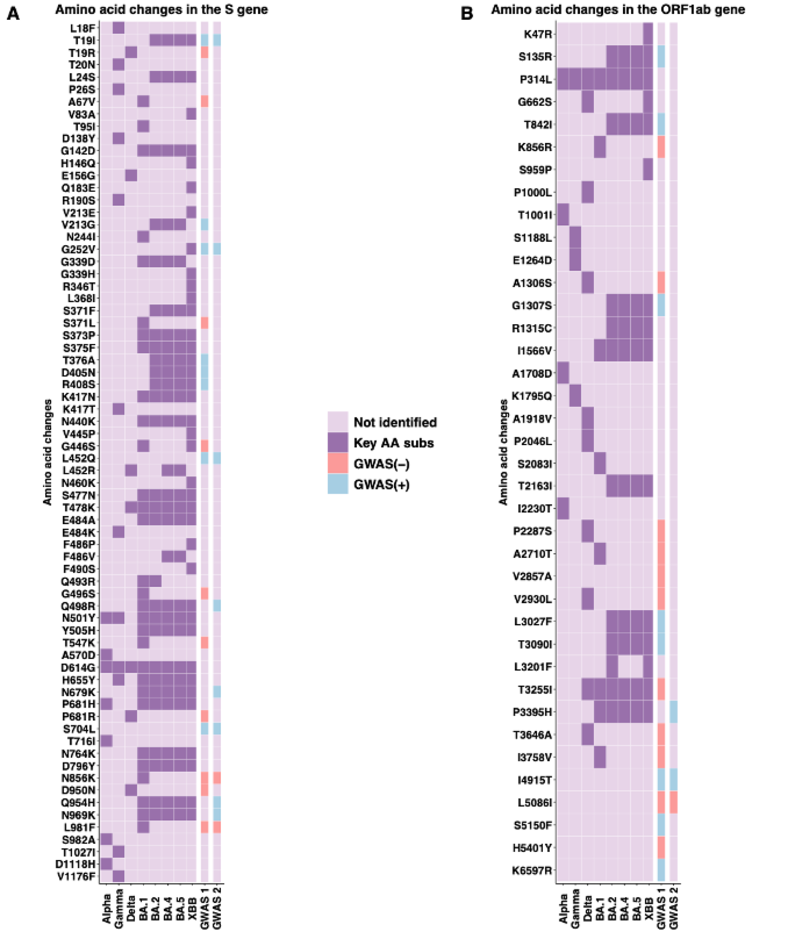

Supplement: S12 Fig — The comparison of the key amino acid changes (dark purple) in each variant, with GWAS-identified SNPs that were associated with negative (red) or positive (blue) effects on viral copies in the (A) S gene and (B) ORF1ab gene. The results of GWAS analysis using the two dimensions computed by MDS as covariates are shown as “GWAS 1”, and the results of the analysis using the categorical clusters as covariates are shown as “GWAS 2”. The effective sizes of identified SNPs using different population control methods are given in S1 and S2 Tables. (TIF) [file pcbi.1012469.s012.tif]
