# Supplementary material for: Genome-wide association study between SARS-CoV-2 single nucleotide polymorphisms and virus copies during infections
Source: PLoS Comput Biol. 2024 Sep 17;20(9):e1012469. doi: 10.1371/journal.pcbi.1012469 (PMC11432881; doi:10.1371/journal.pcbi.1012469)
Supplement: S1 Table — (DOCX) [file pcbi.1012469.s013.docx]

| AminoAcidChange | coefficients | Standard deviation |
| --- | --- | --- |
| T19I | 0.2123842 | 0.03172457 |
| R203M | 1.6492504 | 0.15825678 |
| G252V | 1.2301542 | 0.12381144 |
| L452Q | 0.3127277 | 0.03308999 |
| Q498R | 0.2123842 | 0.03172457 |
| N679K | 0.2123842 | 0.03172457 |
| S704L | 0.3077803 | 0.03301503 |
| N856K | -0.2832514 | 0.03621949 |
| Q954H | 0.2123842 | 0.03172457 |
| N969K | 0.2123842 | 0.03172457 |
| L981F | -0.2832514 | 0.03621949 |
| P3395H | 0.2123842 | 0.03172457 |
| I4915T | 0.3189265 | 0.03351622 |
| L5086I | -1.1958547 | 0.13116867 |
