# Supplementary material for: Genome-wide association study between SARS-CoV-2 single nucleotide polymorphisms and virus copies during infections
Source: PLoS Comput Biol. 2024 Sep 17;20(9):e1012469. doi: 10.1371/journal.pcbi.1012469 (PMC11432881; doi:10.1371/journal.pcbi.1012469)
Supplement: S2 Table — (DOCX) [file pcbi.1012469.s014.docx]

| AminoAcidChange | coefficients | Standard deviation |
| --- | --- | --- |
| D3G | -0.5289821 | 0.06197068 |
| T19R | -0.3583990 | 0.05236202 |
| T19I | 0.8675393 | 0.09368981 |
| S26L | -0.3470353 | 0.05215769 |
| T40I | -0.3810594 | 0.05035466 |
| D63G | -0.3508300 | 0.05236169 |
| A67V | -0.5034788 | 0.06039024 |
| I82T | -0.3456858 | 0.05196310 |
| S135R | 0.8074352 | 0.09059436 |
| V213G | 0.2323215 | 0.03223704 |
| T223I | 0.5872878 | 0.08264916 |
| G252V | 1.1976598 | 0.12468634 |
| S371L | -0.5534597 | 0.06193220 |
| T376A | 0.8620845 | 0.08999643 |
| D405N | 0.8797794 | 0.09241162 |
| R408S | 0.6207915 | 0.08287705 |
| S413R | 0.8157651 | 0.09230739 |
| G446S | -0.2664795 | 0.03198609 |
| L452Q | 0.3466409 | 0.03292778 |
| G496S | -0.5499511 | 0.06210286 |
| T547K | -0.5341148 | 0.06147332 |
| P681R | -0.3506782 | 0.05225140 |
| S704L | 0.3418159 | 0.03284982 |
| T842I | 0.7544330 | 0.08850513 |
| N856K | -0.5521347 | 0.06227279 |
| K856R | -0.5393076 | 0.06198051 |
| D950N | -0.3534498 | 0.05196289 |
| L981F | -0.5521347 | 0.06227279 |
| A1306S | -0.3553043 | 0.05035843 |
| G1307S | 0.8259626 | 0.08905459 |
| P2287S | -0.3541119 | 0.05015436 |
| A2710T | -0.5450373 | 0.06223708 |
| V2857A | -1.3198257 | 0.20083279 |
| V2930L | -0.3796890 | 0.05042589 |
| L3027F | 0.8277260 | 0.08812009 |
| T3090I | 0.7634935 | 0.08992720 |
| T3255I | -0.2507549 | 0.03705032 |
| T3646A | -0.3687172 | 0.05037977 |
| I3758V | -0.5466932 | 0.06187469 |
| I4915T | 0.3524137 | 0.03336264 |
| L5086I | -1.2284741 | 0.13159986 |
| S5150F | 0.8465102 | 0.09162000 |
| H5401Y | -0.3689352 | 0.05186462 |
| K6597R | 0.8501061 | 0.09021831 |
